# The FUNG-GROWTH database: linking fungal phenotype to genome

**DOI:** 10.1128/mra.00378-25

**Published:** 2025-08-06

**Authors:** Ronald P. de Vries, Ad Wiebenga, Guillermo Aguilar Osorio, Maria Victoria Aguilar Pontes, Evy Battaglia, Tiziano Benocci, Isabelle Benoit Gelber, Ourdia Bouzid, Sara Casado Lopez, Nancy Coconi Linares, Alfons J. M. Debets, Jan F. J. de Jong, Elodie Drula, Daniel Falkoski, Jan Grijpstra, Birgit S. Gruben, Sylvia Klaubauf, Claire Khosravi, Joanna E. Kowalczyk, Roland S. Kun, April J. Liwanag, Ronnie J. M. Lubbers, Eline Majoor, Sakinie Misiedjan, Astrid Müller, Şebnem Ozturkoglu Budak, Aleksandrina Patyshakuliyeva, Diogo Robl, Blanca Trejo-Aguilar, Joost van den Brink, Yoni Visser, Alexandra Vivas-Duarte, Aniek Vugts, Miaomiao Zhou, Mao Peng, Pedro M. Coutinho, Bernard Henrissat, Vincent Robert

**Affiliations:** 1 Fungal Physiology, Westerdijk Fungal Biodiversity Institute 141042, Utrecht, the Netherlands; 2 Microbiology, Utrecht University 8125, Utrecht, the Netherlands; 3 Laboratory of Genetics, Wageningen University 593528 , Wageningen, the Netherlands; 4 Architecture et Fonction des Macromolécules Biologiques (AFMB), CNRS, Aix-Marseille Université, INRAE, UMR 1163, Biodiversité et Biotechnologie Fongiques 128791, Marseille, France; 5 Department of Biotechnology and Biomedicine, Technical University of Denmark 5205, Kgs. Lyngby, Denmark; 6 Bioinformatics, Software Development and Databasing, Westerdijk Fungal Biodiversity Institute 141042, Utrecht, the Netherlands; University of California Riverside, Riverside, California, USA

**Keywords:** fungi, growth profiles, monosaccharides, polysaccharides, plant biomass

## Abstract

The utilization of plant biomass, polysaccharides, and associated monosaccharides is a crucial part of fungal physiology. With an increasing number of genomes, annotation can address differences in this process but often lacks detailed biological support. FUNG-GROWTH (https://www.fung-growth.org/) aims to provide and host this to improve genome analysis.

## ANNOUNCEMENT

**Fig 1:**
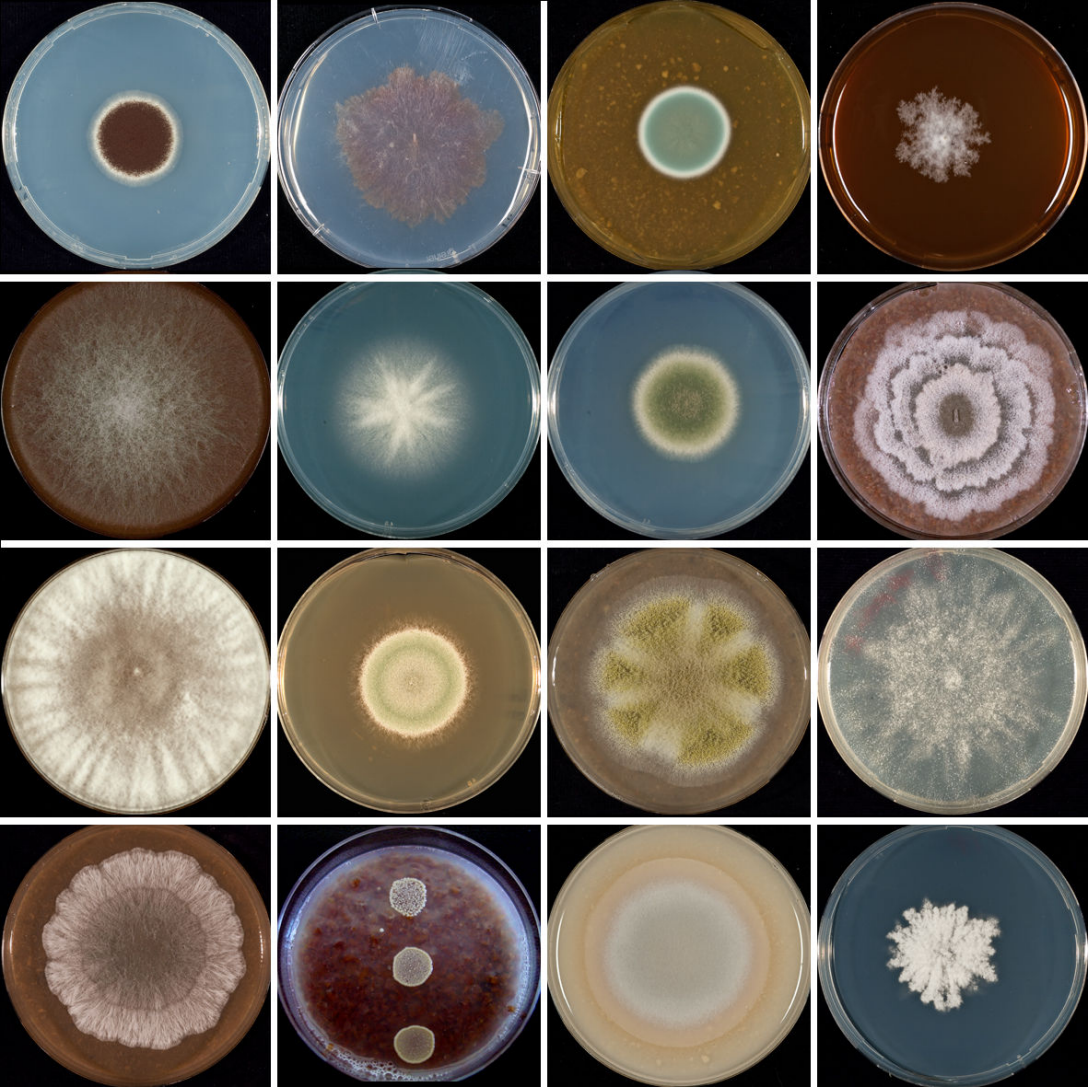
Example of the macromorphology diversity of fungi.

Plant biomass is a major resource for the sustainable circular economy, and fungi have an important role in its biorefinery (1). With an increasing number of fungal genome sequences available and automatic annotation of their carbohydrate-active enzymes (2) on the JGI Mycocosm portal (3), it is becoming easier to do genome-based comparison of the potential of fungal species for plant biomass valorization. However, a genomic potential is not always a direct reflection of a fungus’ ability or behavior, due to gene regulation and other factors, as has been demonstrated in a number of fungal genome studies (4–8). To provide physiological support for the genomic potential, we have performed growth profiles of ~400 fungal species, most with a public genome sequence, on a set of monosaccharides, oligosaccharides, polysaccharides, and crude plant biomass. As fungi cannot import polysaccharides into the cell, the growth on these latter substrates provides insights into their extracellular enzymatic abilities, while the growth on the mono- and oligosaccharides provides insight into their metabolic abilities. The database allows comparative analysis of the fungi by using D-glucose and no carbon source as internal positive and negative controls, respectively, and by comparing the relative growth of another carbon source to these control conditions between fungi. In addition, it demonstrates the influence of the carbon source on fungal macromorphology (Fig. 1).

Growth profiles were generated using 10 monosaccharides (D-glucose, D-fructose, D-galactose, D-mannose, L-rhamnose, D-xylose, L-arabinose, D-ribose, D-galacturonic acid, and D-glucuronic acid), five oligosaccharides (cellobiose, maltose, lactose, sucrose, and raffinose), 11 polysaccharides (cellulose, starch, inulin, beechwood xylan, birchwood xylan, oat spelt xylan, guar gum, Arabic gum, apple pectin, citrus pectin, and arabinogalactan), seven crude plant biomass substrates (wheat bran, sugar beet pulp, citrus pulp, soybean hulls, rice bran, cotton seed pulp, and alfalfa meal), and three non-sugar conditions (no carbon source, casein, and lignin). All carbon sources were added to a minimal salt medium that was shown to be suitable for the species. For sporulating fungi, plates were inoculated with 2 µL of a 500 spores/μL fresh spore suspension in the center of the growth profile plates. For non-sporulating fungi, a ~1 mm stab from the periphery of a freshly grown plate with rich medium (e.g. malt extract agar) was placed on the center of the growth profile plates. The fungi were cultivated in duplicates until the fastest growing colony did not yet reach the edge of the plate. No significant variation in growth on the duplicate plates was observed. A representative picture was taken from each carbon source for each fungus and added to the database. For this, we used a Nikon digital camera D5100 with an AF Micro Nikkor 60 mm (1:2.8D) lens and automated settings. The plates were placed on black velvet, and a circular FalconEyes FLC-40 fluorescent light was used to prevent shadows from the Petri dish edges.

The Fungal Growth database and associated website (https://www.fung-growth.org/) were created using BioloMICS (Bioaware, Hanut, Belgium) (9). Currently, the database contains 398 fungi covering a large part of the width of the fungal kingdom. The database can be searched through its public website (https://www.fung-growth.org/), which also lists recipes of the different minimal media used.

## Data Availability

The database can be accessed at https://www.fung-growth.org/.
